# Dementia Care in Indonesia: Care Networks, Awareness & Perception

**DOI:** 10.1007/s10823-025-09544-x

**Published:** 2025-10-13

**Authors:** Eva S. van der Ploeg, Yvonne S. Handajani, Elisabeth Schröder-Butterfill

**Affiliations:** 1 Department of Gerontology, Faculty of Social Sciences, University of Southampton, Southampton, SO17 1BJ UK; 2 Faculty of Medicine and Health Sciences, Atma Jaya Catholic University of Indonesia, Jakarta, 12930 Indonesia

**Keywords:** Dementia, LMIC, Caregivers, Awareness, Family care

## Abstract

Estimates of dementia prevalence in Indonesia widely vary between studies (9–28%), and little is known about the awareness and perception of dementia and the care networks around those living with (suspected) dementia. This study aims to explore dementia care and awareness in Indonesia. We combine a review of the scientific literature with observational and interview data from an ethnographic study across five sites in Indonesia. We found that awareness of dementia and resources for dementia-specific care are limited in Indonesia. Daily care for people with dementia is performed mainly by family carers with little support. Professional dementia care in Indonesia is rarely mentioned, but a small proportion of the population can access knowledgeable volunteers. Awareness of dementia is low in our study sample, and numerous misconceptions about dementia exist. It is widely perceived as part of normal ageing, and consequently many people with suspected dementia function well, stay active and connected within the community. They are often not recognized as having a health- or cognitive issue, but receive physical, ADL or material care in their homes. When dementia affects people’s behaviour or personality, they may be stigmatized or kept behind locked doors. Recommendations include increasing awareness of dementia symptoms in general and associated unmet needs, as well as a more inclusive style of caregiving and drawing on strengths of Indonesian social structures.

## Introduction

This article explores the care networks of people living with (suspected) dementia in Indonesia and the awareness and perception within these networks of what dementia is and how people with dementia should be treated and supported. Alzheimer’s dementia and related dementias (ADD) affect an estimated 55 million people worldwide. Of those, 60% live in low- and middle-income countries (LMICs) (World Health Organization, 2023). ADD are a major cause of disability and dependency for older adults, impacting not only the person with dementia, but their families, communities, and wider society. The need for support often starts as the person with dementia loses essential life skills with progressive declines (Prince et al., 2013). Consequently, family carers experience ever-changing challenges, including practical (hours caring), psychological (emotional strain, depression, anxiety), and economic (increased expenditure, reduced worktimes) (Prince et al., 2015). Research suggests that negative effects on family carers in LMICs, such as Indonesia, are larger (see Literature Review).

Indonesia is a middle-income country in Southeast Asia and the fourth most populous country globally. People aged 60 and over are considered to be of “advanced age” (*lanjut usia*, shortened to *lansia*). In 2020, 11% of the Indonesian population was aged 60 and over, and this is expected to increase to 20% by 2045 (Badan Pusat Statistik, 2021). Over a decade ago, Kadar et al. (2013) observed that Indonesian healthcare for older people was only gradually becoming a priority. The authors described a lack of healthcare services for older community-dwelling people, partly due to personnel shortages, including community nurses. Indonesia has few long-term care facilities (Mahendradhata et al., 2017; Asmorowati et al., 2024), suggesting that, as in many LMICs, dementia care in Indonesia is mainly performed in the community by family members. As described by Mahendradhata et al. (2017, p.164): “It is part of Indonesian culture that family members become informal carers of the elderly […] Respite care for informal carers has not yet been developed.”

Estimates of dementia prevalence amongst Indonesians aged 60 and over vary widely. In 2015, Alzheimer Disease International estimated that 1.2 million Indonesians (4% of the 60 + population) lived with dementia (Prince et al., 2015). Hogervorst et al. (2021) estimated 9% of older people lived with dementia. However, some estimates are much higher, ranging from 20% to nearly one-third of older Indonesians (Farina et al., 2023; Handajani et al., 2024; Suriastini et al., 2020). These percentages lie well above global estimates (4.6–8.7% of those aged 60 and over, Prince et al., 2015) and warrant careful consideration. We notice some variety in sample age: Farina et al. (2023) included people aged 65 and over (dementia prevalence of 28%), however both other Indonesian-based studies included people aged 60 and over. Some dementia measurements are not considered equally appropriate in all settings. For example, Suriastini et al. (2020) used the MMSE, which can be challenging to implement in low-education populations and has cross-cultural issues, and Handajani et al. (2024) analysed survey data based on a noun recall question and self-reported memory issues. However, Farina et al. (2023) used instruments fitting cross-cultural settings. Importantly, these prevalence data are based on survey data rather than formal diagnoses. Farina et al. (2023) showed that only five of their 2,110 study participants (0.2%) had received a dementia diagnosis. Based on the existing evidence, we can only conclude that prevalence of dementia for older Indonesian people (60+) is likely to be in the range between 9 and 28% and requires further study with region-appropriate measurements. Despite these potentially high levels of suspected dementia, public understanding of dementia is low: in a survey amongst 4,430 members of the general public, 86% of respondents had not heard of ADD and viewed it as a normal part of ageing (Farina et al., 2024).

In terms of formal dementia support in Indonesia, the Alzheimer’s Indonesia foundation (AlzI) was established in 2013, and the Health Ministry initiated the country’s National Dementia Plan (NDP) in 2016. AlzI is the only nation-wide organization focused on dementia. Of AlzI’s eighteen national chapters, sixteen are located on the three most populous islands (Alzheimer’s Indonesia, n.d), specifically in large cities (0.5–10.5 million people). The remaining two chapters are on smaller, less populated islands. Even if all inhabitants of cities and islands with AlzI chapters were aware of their existence and sought them out, an estimated 88% of Indonesians do not have access to dementia-specific services. This estimation ignores that the proportion of older people living in rural areas is significantly higher than in urban areas (Kadar et al., 2013). AlzI and the NDP focus on increasing awareness and developing programmes for dementia education, yet recent evaluations question the success and reach of these awareness campaigns (Farina et al., 2024; Suriastini et al., 2020).

We aim to explore dementia care and awareness in Indonesia. We review published literature before drawing on recent empirical data. Using qualitative data from an anthropological study across five sites in Indonesia, we describe the care networks surrounding older people living with dementia and carers’ awareness and perceptions.

Our research questions are:

1. Who cares for people with dementia in Indonesia? What kind of care is provided, and how is caring perceived?
2. What is the level of awareness of dementia and how is dementia described by carers?

## Literature Review

Given potentially elevated levels of dementia and under-developed care infrastructure resulting in little dementia support and knowledge, we explored what evidence exists about dementia care networks in Indonesia and the awareness and perception of dementia in these networks. Emergent research evidence from Indonesia reveals that typical family carer challenges and burdens apply, similar to other LMICs (Turana, 2022). The challenges for family members in LMICs are often larger for reasons beyond relatives being the primary (and/or sole) carer. Firstly, in addition to limited public health infrastructure for dementia, public awareness and education about dementia are lower (Dominguez et al., 2021; Mushi et al., 2014; Wang et al., 2014). Secondly, cultural narratives of collectivism and family involvement may lead family carers to feel obliged to care without seeking professional help (Wang et al., 2012). In Indonesia specifically, barriers to good care include family carers’ limited dementia knowledge and -skills; low awareness in the community, and limited availability of dementia specialists (Widyastuti et al., 2023). Focus group discussions with 19 family carers of people with dementia showed that families are providing care in an environment that lacks understanding, which can lead to misdiagnosis and feelings of fear and shame (Theresia et al., 2023).

However, caring may have positive aspects and consequences as well. A worldwide systematic review of 81 studies with 3,347 participants concluded that positive experiences of caring for family carers include personal accomplishment and strengthened relationships (Devi et al., 2020). In Indonesia, existing social structures may add additional positive aspects to caring. Widyastuti et al. (2023) describe that support received from close and distant family members reduces carers’ burden and improves quality of care. Other Indonesian studies reveal that positive aspects of dementia caring include experiencing gratitude and closeness to the person, increased patience and resilience, and establishing meaningful, affectionate relationships (Kristanti et al., 2018; Pradana et al., 2021).

Few studies have explored attitudes and beliefs around dementia in Indonesia. The 2019 World Alzheimer Report focused on worldwide attitudes towards (people living with) dementia (Lynch, 2020). The report presents a survey conducted in 155 countries (including Indonesia), with approximately 60,000 respondents, including 1,446 people living with dementia, 18,377 carers for a person living with dementia, 14,124 healthcare practitioners, and 26,913 members of the public. The global report zooms in on countries where national findings either confirm or differ significantly from global trends. It revealed that a large proportion of the Indonesian public (44%) believed that people with dementia were dangerous. This percentage was higher amongst Indonesian healthcare practitioners (66%). Three-quarters of the Indonesian public thought people with dementia were unpredictable and impulsive, and 19% of the Indonesian public and 20% of healthcare practitioners stated that they would keep a dementia diagnosis secret.

Farina et al. (2024) reported in more detail on the Indonesian general public sample (*n* = 4,430) included in the worldwide survey. Most Indonesians (86%) had not heard of the term dementia or Alzheimer’s disease and commonly viewed dementia as a normal part of ageing. Three quarters describe people with dementia symptoms as being *pikun* (senile). Combining the global and national reports, it seems that the Indonesian public is largely unfamiliar with dementia, but holds negative perceptions of those living with it, namely that they are unpredictable, impulsive and dangerous. Views of Indonesian healthcare practitioners were barely reported and showed that their perceptions of (people with) dementia were more negative than those of the general population.

In a study conducted in Yogyakarta (*n* = 203), participants mostly viewed Alzheimer’s disease as “pessimistic” (Mulyani et al., 2019). Widyastuti et al. (2023) reported that family carers face discrimination within the community when dementia is labelled a psychiatric condition. In this study, the community’s stigma towards people living with dementia was manifested through labels such as “crazy” or “insane” to describe them. Despite some studies reporting both positive and negative aspects of caring for a relative with dementia, reported attitudes towards dementia are mostly negative.

In summary, awareness of dementia in Indonesia is low, with a general local descriptor of *pikun* (senile) being applied to older people whose functioning changes over time. The existing scientific research on Indonesian care networks is limited and our descriptions are mainly based on national policy descriptions. However, we notice a trend across sources that family carers are the first and often only support for people living with dementia and fulfill all necessary roles. Studies focusing on the perception of (taking care of) people living with dementia reveal considerable variety between samples. Caring for a family member has both positive and negative aspects and consequences, but overall attitudes towards dementia seem negative. From the limited sources available, it seems that both the Indonesian public and health care practitioners frequently take a negative stance, describing people with dementia as dangerous, unreliable or crazy. This anthropological study specifically aims to provide more insight into who cares for people with dementia and in what manner, a topic that has not been explicitly studied until now. In addition, it explores awareness and perception of these dementia carers.

## Methodology

### Study Design

This paper is based on a research project titled “Care networks in later life: A comparative study of five communities in Indonesia”, which ran from 2019 to 2023.^1^ This project examined the care needs and care networks of older people (60+) in five communities across Indonesia, using an ethnographic, qualitative approach. The communities include a matrilineal highland village on Sumatra; an impoverished, densely populated multi-ethnic neighbourhood of Jakarta, the country’s capital located on the island of Java; further peri-urban Javanese sites in Yogyakarta and East Java; and a fishing and farming community on a remote island of East Nusa Tenggara. All but the Yogyakarta site had been studied by members of the research team before, which facilitated the recruitment of care dependent older people. In the sites on Java availability of primary healthcare for older people is good, and volunteer-supported community health initiatives occur, whereas on the remote island and in the Sumatran community distances to health providers are greater and initiatives for older people lacking.

### Participants & Recruitment

The *Care Networks* project identified 10–12 care-dependent older people in each of the five study communities, drawing on previous familiarity with families and recommendations from local community leaders. Inclusion criteria were being aged 60 and over and having a need for care. We sampled for a range of care needs (assistance with instrumental or basic activities of daily living, mobility limitations, being blind or deaf, having cognitive impairment). For each care dependent older person, we also recruited one to five members of their care network. In addition, we conducted interviews with healthcare volunteers (kader). *Kader* are local volunteers who support community-based health professionals in the delivery of health-outreach activities, especially monthly health checks for older people (Pratono & Maharani, 2018). *Kader* are not medically trained, nor have they received any specific training about dementia. We also interviewed three volunteers from Alzheimer’s Indonesia (AlzI). Three of our field sites (Yogyakarta, Jakarta and Malang) are located within the operating sphere of an AlzI chapter.

We emphasise that this paper, which focuses on (suspected) dementia, draws on a small subset of data from the Care Networks project, because most care dependent older people in our study did not show signs of cognitive impairment. There were eight people referred to by family members or kader as being *pikun* (local term for suspected dementia) or displaying behaviours suggestive of cognitive decline. None of our study participants had a dementia diagnosis, hence our use of ‘suspected dementia’. For the present analysis, we draw on data from interviews and observations with family carers of the eight older people with suspected dementia; interviews with ten community health volunteers (*kader*) who discussed dementia; and three volunteers working for AlzI (demographic details in Table 1). As the number of participants with dementia-relevant information in each site is small, we are not drawing cross-site comparisons.

**Table 1:** Demographic details for family members and volunteers included in the study

Data report #	Role (*n*)	Region	Socio-economic status	Age group	Gender	Relationship to person w/dementia
FM1	Family carers (2)	East Java	Higher	40–49, 30–39	2 Females	Adult daughter and daughter-in-law
FM2	Family carer	Sumatra	Lower	40–49	Female	Adult niece
FM3	Family carer	Jakarta	Higher	50–59, 70–79	Female	Daughter and wife
FM4	Family carers (3)	East Java	Lower	30–39, 50–59, 50–59	2 Females and 1 Male	Granddaughter, daughter and son
FM5	Family carers (3)	East Java	Higher	40–49, 60–69, 40–49	3 Females	Daughters and daughter-in-law
FM6	Family carer	Sumatra	Lower	80–89	Male	Husband
FM7	Family carer	East Nusa Tenggara	Lower	30–39	Female	Daughter-in-law
FM8	Family carer	Jakarta	Lower	50–59	Female	Daughter
ALZV1	AlzI volunteer	Yogyakarta	-	30–39	Female	-
ALZV2	AlzI volunteer	Yogyakarta	-	40–49	Female	-
ALZV3	AlzI volunteer	Yogyakarta	-	40–49	Female	-
KADER1	Kader	Jakarta	-	60–69	Female	-
KADER2	Kader	Jakarta	-	50–59	Female	-
KADER3	Kader	Jakarta	-	30–39	Female	-
KADER4	Kader	Jakarta	-	50–59	Female	-
KADER5	Kader	East Nusa Tenggara	-	30–39	Female	-
KADER6	Kader	East Nusa Tenggara	-	30–39	Female	-
KADER7	Kader	East Nusa Tenggara	-	40–49	Female	-
KADER8	Kader	Yogyakarta	-	30–39	Female	-
KADER9	Kader	Yogyakarta	-	30–39	Female	-
KADER10	Kader	Yogyakarta	-	20–29	Female	-

### Data Collection

Ethnographic fieldwork was undertaken by Indonesian anthropologists with relevant local language skills, supported during regular team meetings by the wider research team who speak the national Indonesian language. Data collection included in-depth repeat interviews using semi-structured interview guides; and participant observation in participants’ homes and during community health events. Interviews with family caregivers covered the history of the relationship with the older person, nature of care needs and caregiving tasks, division of labour within family networks, positive and challenging aspects of caregiving, older person’s health and healthcare use. Observations focused on care needs, caregiving, and interactions between care network members. Interviews with volunteers probed, among other things, their awareness and experience of dementia, understanding of dementia aetiology and progression, terminology used, and characteristics ascribed to people with cognitive decline, treatment of people with suspected dementia (both in terms of healthcare and socially).

### Analysis

Interviews were audio-recorded, and the anthropologists took notes during conversations and observations. On this basis, detailed fieldnotes (rather than interview transcripts) were written up in Bahasa Indonesia, with verbatim extracts added where exact wording was important. For this paper, the empirical basis consists of all observation- and interview notes relating to the eight older persons with suspected dementia and the interview extracts with the volunteers that relate to dementia. We used qualitative content analysis by extracting data in MS Word under broad pre-determined topics (e.g. awareness, perception, care approach, professional support), and then inductively developing codes to capture differences and nuances of responses (e.g. disempowering care; stigmatisation; tolerance). These were then examined further to check for patterns (e.g. volunteers versus family carers; respondents with more versus less knowledge about dementia).

When data extracts are included in the results section below, we clarify in (round) brackets whether they are taken from the anthropologists’ summaries of interviews and observations (e.g., fieldnote Kader2) or represent verbatim interview extracts (e.g. Kader2); quotes are also indicated by quote marks.

## Results

### Care Networks

In line with findings from the literature, we found that care for people with suspected dementia in Indonesia is primarily the responsibility of family members. More specifically, the primary carer is usually female and often the spouse or daughter (in-law) who was already living with the person or who moved in since cognitive problems emerged. In one case, a husband was caring for his wife, because the couple had no children. While none of the older people with suspected dementia lacked a primary carer, there were sometimes shifts in the identity of the primary carer because of other priorities or needs arising. This underlines the fact that family carers often juggle multiple roles, including paid work (which may necessitate prolonged absence from home), raising children or grandchildren, housework, and care for one or more older family members. As our data collection focused on older people and the caring network around them, we were able to examine division of labour within families. Many, but certainly not all, primary carers receive assistance from other relatives or neighbours or occasionally from paid domestic helpers in better-off families. Our evidence suggests that larger family networks are at an advantage, allowing primary carers to delegate certain care responsibilities. For example, one well-to-do coresident daughter in our Sumatran site continued working as a teacher because her unemployed son was able to stay with his grandmother during the day. By contrast, an impoverished single woman in the same community, who was caring for both her paralysed mother and aunt with suspected dementia, resorted to locking her relatives in the house when she went out to work because she lacked siblings and children. Her aunt’s sons had distanced themselves from their mother on account of her smell and erratic behaviour. Thus, variation in the size and wealth of care networks influences the quality and sustainability of dementia care.

*[She] said that none of her relatives helped. [She] said that if someone’s situation is difficult [referring to financial burdens of caring]*,* then no relatives […] will approach. [She] said that because of her difficult situation*,* her siblings were afraid that she would ask for help. […] [She] added that there were also (distant) relatives who were willing to help and were kind. (Adult niece caring for aunt (fieldnote FM2))*

Unless cognitive decline affects a person’s ability to manage activities of daily living and stay safe, it is not considered necessitating care, treatment or surveillance: the older person is ‘let be’. As needs rise, family members’ care tends to focus on hands-on tasks, like providing food, facilitating health-centre visits, washing, grooming and toileting.

*To meet the needs of [older woman with symptoms of dementia]*,* especially food intake*,* the children around [her] house ([names of four children]) take turns preparing food three times a day. […] The Bali and Jakarta daughters … provide relatively regular monetary support. (Adult daughter and daughter-in-law caring for older woman (fieldnote FM1))*

Most family carers have not received or sought out education regarding dementia and are unfamiliar with the syndrome and how best to interact (see Awareness & Perception, below). Day-to-day care is described in a way that suggests that the style of caregiving is one of taking over and excluding the person with suspected dementia. One AlzI volunteer mentioned that often family members (and volunteers alike) talk about the older person whilst they are in the room, yet do not include them in the conversation. When the person with suspected dementia makes mistakes, they are likely to be prohibited from performing the activity again, and someone else takes over. Extended family and those living far away may provide financial or moral support but sometimes are not involved at all. We encountered several cases where relatives ceased visiting the older person with advanced symptoms of dementia, because this was considered in vain. Support from family members living in proximity is augmented by ad hoc assistance from unrelated community members, who may for example keep an older person company while the primary carer is out.

*Nearly setting the kitchen on fire has resulted in the intervention that [the older woman with memory problems] is not allowed to cook any more. Losing money has resulted in the granddaughter giving her basic supplies instead of money. (Wife and adult daughter caring for older woman (fieldnote FM4))*

*[Woman with suspected dementia] has become terribly thin over the last few months*,* and recently she fell out of the window because she tried to climb out when locked in. It seems she would prefer still to roam around*,* but the presence of her daughter prevents this. (Adult daughter and daughter-in-law caring for older woman (fieldnote FM1))*

Care or support from professionals, much less from dementia care experts, is rarely mentioned in interviews and when it is, it is labelled as missing. Although in four of our five sites general health checks for older people are held regularly, they are exclusively focused on physical health (e.g. blood pressure and sugar measurement). In one community clinic in Yogyakarta a “dementia corner” is mentioned: it provides dementia-specific information and materials. The sole resource of support outside family or community members we identified, were volunteers, either affiliated with the national organization of Alzheimer’s Indonesia or *kader*. However, these volunteers clearly state that it is not their job to label or diagnose people; all they can do is recommend to family members that the older person visit a healthcare professional. Actual referral to physicians seems to happen infrequently and solely for the purpose of receiving a diagnosis. In those few cases, physicians do not always provide clarity or follow-up. Altogether, engagement with healthcare services among older care dependent people across our study sites is low, and even more so among older people with suspected dementia. According to our interviews, some individuals even actively avoid medical care and refuse to see a doctor for several reasons, including financial- and time constraints, problems with transportation, and a reluctance to receive a diagnosis or treatment.

*“We are just kader*,* not health workers. We can’t do tests like that. At most we can only give advice*,* just suggest it to their children. We talk to the children slowly*,* so that the older relatives are taken to the doctor.” (Kader4)*.

*“Although older people who have cognitive problems often cause headaches for kader and nurses*,* they have never been referred to the community health centre so far.” (Kader6)*.

*[Woman with suspected dementia] is never taken to the hospital*,* because the primary carer feels it is more important to help her husband to secure income. (Daughter caring for older woman (fieldnote FM8))*

Thus, the main support for family carers is volunteers, where available. Both *kader* and AlzI volunteers mention that in addition to advising people to visit physicians, they consider their main job to provide moral support. The Yogyakarta AlzI chapter has structured activities aimed at people with (suspected) dementia, and *kader* interviewed in Yogyakarta mentioned both receiving some training and guidance about dementia as well as offering some ‘brain training’ exercises. In terms of how volunteers communicate, observers repeatedly recorded that volunteers do not speak to the older person directly, but to family members. The amount and type of support offered seems to rely on a few passionate and knowledgeable individuals supported by others with little dementia education or experience, who are more passive.

*“Carers who are members of AlzI Yogyakarta have a communication medium in the form of a WhatsApp Group. In the WhatsApp Group there are also doctors and psychologists. Conversations in the WA Group can become lively because carers have almost the same problems and then share them with one another.” […] [She] explained that the support through home visits that she has been doing so far is more moral support. So far*,* she has provided moral support by coming with food and listening to the carers [pouring out their] hearts. (fieldnote ALZV3)*

*[She] stated that the kader could only listen to [the confiding] by the older carers and [could not take away the burden] because it was impossible for them to go deeper into managing the emotional relationship in the family between the senile elder and their carers. (fieldnote Kader2)*

*“I tend to be passive because I feel that I do not really understand dementia in theory. […] Usually everything [is guided by] [ALZV3]. […] As soon as [she] fell ill*,* AlzI Yogya ceased to function.” (ALZV2)*.

Where the literature mainly revealed that family members are the main resource of dementia care, data from this study provide more detail about which family members provide care, whether they have a surrounding care network, who this consists of and what kind of care these individuals and networks provide. In summary, care for people with dementia falls on one primary family carer, who may or may not have support of other family members (near or far), the wider community, paid professionals and/or volunteers with general medical knowledge (*kader*) or some dementia-specific skills (AlzI). Primary carers perform most of the care tasks, including the physically more strenuous ones such as personal care. Other family members may also be involved with these tasks, but some only provide monetary support (especially when living elsewhere). Volunteers, if they are involved at all, mainly offer moral support and advice. Only in our Yogyakarta fieldsite did we encounter dementia-specific facilities being developed via AlzI, although we should note that this was the only site where AlzI volunteers were interviewed. Reports about dementia specific activities in other regions were shared by neither family members nor *kader*.

#### Awareness and Perception

Having established that family members and volunteers are the “dementia care network” in our study sample, we wanted to explore how these carers perceive (people with) dementia. Based on our observations and interviews with family members and volunteers, it is evident that awareness of dementia, cognitive decline and memory loss is low. The word “dementia” was never used, and we were either drawn to a case because there was mention of *pikun* or because our team members recognized symptoms that could point to dementia. Family members spontaneously describe dementia symptoms in terms of incidents, changes, or problems, rarely linking them to an underlying cause or to brain functioning. Examples include their reduced ability to operate household appliances; repeating sentences or questions; hoarding or hiding food; forgetting they had eaten; misplacing or overspending money; trouble sleeping or with personal hygiene; and behavioural changes.

*“[She] is shabby and tends to smell*,* [this] is because she rarely bathes or has difficulty bathing and when she does*,* it’s only moderate. In addition*,* the clothes worn are rarely changed even though they have been worn all day and are exposed to sweat.” (Adult niece caring for aunt (FM2))*.

*“He is no longer able to hold his urine back and also has a returned ‘pleasure of seeing beautiful women’.” (Wife and adult daughter caring for older man (FM4))*.

These quotes illustrate carers’ picking up on changes relating to core capabilities that adults normally have but which are deteriorating in people with dementia. Regular bathing (normally twice daily in rural Indonesia), for example, is socially expected and not readily delegated. Perception of people with suspected dementia is similar across interviewees except for some individuals who have actively deepened their knowledge of dementia. Most participants describe symptoms of suspected dementia as either a normal part of ageing or being *gila* (crazy). When suspected dementia is labelled as normal ageing (*sakit tua*), symptoms are first and foremost attributed to physiological ageing. People with early or mild signs of cognitive impairment are often left to their own devices and allowed to roam freely and participate in the community. This enables continued activities and socialization.

*[She] stated that in general she observed that ‘senile’ (pikun) older people were actually loved by the local residents. If a ‘senile’ older person sits alone*,* there will definitely be local residents who will come over and give him food. (fieldnote Kader2)*

Sometimes the ‘letting be’ of people with dementia has more negative connotations, and people become isolated or are left alone “because they will die soon,” as an AlzI volunteer put it. Isolation and neglect happen more frequently when a person with suspected dementia is seen as ‘crazy’. This interpretation of someone’s behaviour follows from a person acting out of the ordinary or erratically. For example, one *kader* reported on a man who chased visitors holding a knife when they entered his property. The comparison with children is made frequently, even by those who received dementia education.

*For several weeks*,* [he] often heard [his wife] as if she was dreaming*,* she looked out and saw people sitting there*,* even though the ‘people’ that [she] saw were chickens. [She] even saw someone sitting in her bedroom […]. This is what made [him] ask his wife not to leave the house. (Husband caring for older woman (fieldnote FM6))*

*[She] regrets that today’s children … laugh at ‘senile’ older people. She added that children also often judge older and ‘senile’ people as ‘crazy’ because they sometimes wander around and forget to go home*,* so the family usually forbids them from going out*,* only staying at home. (fieldnote Kader6)*

*[Volunteer] then told me that caring for older people and young children is actually almost the same. For [her]*,* taking care of small children is even more tiring. What she found when she cared for older people was that they slept more than children. (fieldnote ALZV2)*

These data extracts underline participants’ predominantly benign perceptions of individuals with dementia (dream-like, like children, sleeping), yet also how these perceptions justify making decisions on their behalf and restricting their movements. Sometimes dementia is perceived or diagnosed as something else, for example hearing or vision impairment. One family carer was told in hospital that her father suffered from “brain stem death”. Whilst frequently dementia is not recognized, the opposite also happens where other physical or mental health problems are labelled as dementia. This is well illustrated by a family member’s description of an older woman from East Nusa Tenggara as being *pikun* (senile). However, the narrative suggests that the behavioural problems the woman was displaying stemmed from unsuccessful cataract surgery leaving her blind and upset.

*In addition to experiencing memory loss*,* she also lost the ability to see six years ago. This decreased ability to see occurred since [she] underwent cataract surgery […]. According to the family*,* the results of the operation did not improve her vision but instead caused blindness. […] Since finishing the operation*,* the older woman became temperamental*,* often shouting and scolding her family. “It’s normal to be angry. […] When she was still healthy*,* she never got angry with me.” (Daughter-in-law caring for older woman (fieldnote FM7))*.

*[She] then gave the example of [resident]*,* who according to her*,* as she gets older*,* her memory about the location of places (spatial memory) becomes increasingly diminished*,* especially when her eyes start not being able to see clearly*,* such as when she has to leave the house in the afternoon or evening. (fieldnote Kader3)*

*“The results of research among students and nurses showed the same results*,* namely that their knowledge about people with dementia was lacking but their attitude towards them was good. […] Students confuse dementia with hearing impairment*,* but it’s impossible for a cognitively normal ex-principal to eat and then spit her food on the floor!” (ALZV1)*.

Different causes of dementia are reported. One of the AlzI volunteers offers online dementia education, where she mixes modifiable risk factors of dementia reported in the Lancet Commission Report (Livingston et al., 2024) with anecdotal and personal ideas, such as a connection between memory loss and the use of aluminium pans, for which scientific evidence is lacking. Those with no formal dementia education attribute the symptoms to causes like social connectivity and loneliness, which is, in fact one of the risk factors for late life cognitive decline described in the Lancet Commission Report.

*“Maybe because older people are not taken care of daily by their families*,* that is the cause of memory loss. Here […] there are no older people like that. Adult children still want to look after them*,* advise them*,* take care of them. … If an older person is left alone most of the time*,* he can get stressed.” (Kader4)*.

*According to [Kader]*,* this dementia is experienced by most people who work and frequently command*,* then suddenly don’t do anything at home: […] “older people with conditions like this just need friends to chat with”. (fieldnote Kader9)*

*“Maybe [she] is angry. We young people are confused. [Woman with suspected dementia] feels that she is not being served as she wants. Or misses her children who are out of town. After her child came [to visit]*,* she [started eating].” (Kader8)*.

Across all interviewees there was agreement that caring for a person with dementia is hard. Family members often experience emotions like guilt, fear, and shame when a person is labelled as crazy. Volunteers frequently mention that family members would benefit from dementia care training. However, often the need for financial support to pay for healthy food and medication is more pressing. More positive attitudes were also reported, mainly psychological support from the community. As illustrated by previous quotes, it is frequently assumed that people with (suspected) dementia need ongoing activities and social contacts, and community members may assist with creating activities and connection.

*“The carer’s difficulties will be faced continuously and for years with dementia which will get worse so that the carer can be more stressed than the patient. Carers who have to [care for] older people with dementia for 24 hours will experience stress because they have to leave their jobs*,* have to lose their social life because those who are looked after will disappear*,* slip and so on.” (ALZV3)*.

*The psychological support that [kader] means is support for community members not to isolate [person with suspected dementia] and not to gossip about him: “Don’t gossip that [he] is senile*,* forgetting his children.” [Kader] further said that if something like that happened it would break the family’s spirit*,* so according to [kader] what the family really needs is psychological support and enthusiasm from the community. (fieldnote Kader10)*

Indeed, as the previous section showed, a combination of poor understanding and economic and social pressures can result in care practices which curtail the older person’s autonomy. However, despite caring generally being challenging, positive stories also emerge.

*“Because [certain family carer] used to be the coordinator of AlzI Yogyakarta*,* she studied continuously*,* and she also educated her children and husband so they would understand the condition of their parent. […] I was impressed with that family. I saw that the [person with dementia] was more beautiful when she was cared for by [family carer] than when she was young.” (ALZV2)*.

Similar to the findings in the literature review, awareness of dementia is low in our study sample, apart from volunteers who have received some dementia education and some individuals who have actively sought out education. Even in those with some dementia education, and more so in those without, misconceptions exist, like confusion about what dementia is and is not; the likening of older people with dementia to children; and misinterpretation of causes of symptoms and how they can be recognised and addressed. These insights into a lack of familiarity and understanding provide a context as to where care can improve, because it is not adequately tailored to include the person with (suspected) dementia and address their specific needs.

## Discussion

Based on findings from the literature review and ethnographic study, we conclude that there is little professional healthcare infrastructure in Indonesia for people living with dementia, and family members deliver most of the care and support. Awareness of dementia is low across societal groups, including medical health professionals. Symptoms are frequently attributed to another disability, such as vision- or hearing impairment, or the other way around, when physiological symptoms are seen as indicators of brain changes. As in many LMICs, a large majority of the population has not heard of dementia as a distinct condition (Johnston et al., 2020). The perception of a person with (suspected) dementia depends on how well the person functions. If symptoms are mild, they are associated with normal ageing and the person is labelled as *pikun* (being senile) or not labelled at all. When symptoms become more pronounced, people may be seen as crazy or dangerous. If people with dementia behave in a relatively conventional or expected manner, they are left to their own devices and allowed to function in the community. Despite a general lack of understanding of dementia, there is a variety of attitudes towards people who have dementia with both positive and negative attitudes and experiences reported.

From the interviews and observational data, we were able to obtain further nuance on these general findings. Most family carers are women, who often juggle multiple roles and have limited knowledge about dementia or even its existence. The size of care networks and type of care they offer vary hugely. One commonality is that every person has at least one informal carer, often wives or daughters (in-law), but sometimes also more distant relatives or community-members. As Sya’diyah et al. (2023) mentioned, in the face of manifest care need there will invariably be someone to fulfil the role. Sometimes one family carer supports a person with suspected dementia alone; yet many primary carers are supported by close family, and some are helped by one or more (extended) family members, near and far, and/or members of the community. Cooking or providing money to buy food is amongst the top priorities, as is maintaining personal hygiene. The style of caring is frequently disempowering by taking over tasks and excluding the person with suspected dementia from engaging in meaningful activities. However, it seems that social exclusion is not frequent, unless someone is seen as ‘crazy’. There are many stories of people actively communicating with people that are perceived as *pikun*.

The support system for family carers consists of volunteers, affiliated with either community health posts for older people (*kader)* or with Alzheimer’s Indonesia. *Kader* are local volunteers with no medical training or dementia-specific knowledge. AlzI employs a few highly skilled individuals, but knowledge, hands-on experience and effort are variable among volunteers. In addition, the focus of AlzI, by their own admission, is to raise awareness rather than provide hands-on care or non-pharmacological interventions. Indonesia being a large archipelago, we estimate that a small minority (at best 12% of the population) can potentially connect with a volunteer who has dementia-specific knowledge. This percentage does not account for the fact that more older people live in rural areas nor that social conventions may prevent people from seeking medical or other support (Kadar et al., 2013; Mahendradhata et al., 2017). This underlines our observation that dementia care is in the hands of family carers who have little to no understanding about dementia nor a connection to someone who does.

However, Indonesian societal structures and conventions also have positive impacts on dementia carers and those living with dementia. Firstly, many primary carers in our sample reported receiving support, for example from (extended) family members, neighbours, or community volunteers. It has been shown that support received from family members reduces carers’ burden and improves the quality of care (Widyastuti et al., 2023). Secondly, problems that carers report when caring for people living with dementia are often phrased in terms of incidents, changes, or problems, rather than linking them to an underlying cause or brain functioning. Our participants rarely consulted with medical professionals, and none had received a formal diagnosis of ADD. This lack of formal diagnosis is often portrayed as negative in the literature. However, the importance of early diagnosis is tempered both by the lack of conclusive evidence supporting medical treatment for ADD, and the general lack of treatment and support for people with dementia in many LMICs. As summarized by Fletcher (2024, p.165), “Given the wealth of evidence pointing to poor efficacy and negative complications [of pharmacological treatments], one might assume that treatment access would be a poor justification for early diagnosis. Nonetheless, it is repeatedly used as a key rationale for diagnosis.” Building on Fletcher’s thought-provoking statement, we believe that dementia symptoms are an easier starting point for non-pharmacological interventions to support people living with dementia than trying to seek and come to terms with a dementia diagnosis. An emphasis on symptoms could help carers select interventions or adaptations that are relevant to the person and how they present each day, which varies greatly between and within individuals (O’Connor et al., 2009; Schwertner et al., 2022). For example, misplacing money can be addressed by having a fixed place where money is kept or a certain person who keeps it safe. Problems with operating household appliances can be reduced by putting up straightforward images of each step on how to use the appliance. These simple adaptations can stimulate people with dementia to maintain function and independence. Finally, the way communities speak about people with dementia reveals their attitudes to them (Swaffer, 2014). People with dementia being seen as ‘ageing normally’ can positively contribute to older people with (mild) cognitive symptoms being allowed to function as before, leading to ongoing social engagement and meaningful activities. While in Western cultures there is a common call for such engagement for those living with dementia, in Indonesia we found evidence of natural inclusion of people who are ageing, even if their functioning, health, personality or behaviour changes. The exception is those who are seen as ‘crazy’ by behaving in unexpected and non-normative ways and are either hidden behind locked doors or ridiculed in the community.

One of the main areas for improvement in current caregiving practices as found in our data is to employ a less disempowering style of caring and communication vis-à-vis the person with dementia. More than two decades ago, Cohen-Mansfield (2004) shared her *unmet needs paradigm* whereby people with dementia behave in an agitated manner when their needs, for example for stimulation, social interaction, and physical comfort, are not perceived or addressed by carers. She postulated that these unmet needs could be tackled via “person-centred” care and psychosocial interventions designed to elicit people’s interest, engagement, and participation. For example, instead of banning a person from cooking because of safety concerns, as was the case in one of our fieldsites, family carers can learn how to include people with dementia using Montessori principles, which suggest dividing tasks into small steps and repeatedly demonstrating what the person is supposed to do (Camp, 1999). Another area for improvement is to challenge attitudes towards people who are stigmatised due to erratic or unusual behaviour. A path to more positive attitudes and support for people with advanced dementia could be through ongoing awareness campaigns. This warrants an evaluation of current initiatives, as it is unclear to what extent awareness campaigns have been rolled out in Indonesia and what their impact is (Farina et al., 2024; Suriastini et al., 2020).

The data for this ethnographic study were collected in five disparate communities across Indonesia with the aim to capture variation in ageing experiences. However, all sites have been involved in ageing research for several years, and three sites were in the vicinity of active AlzI-chapters. This means that even in the more remote sites included in the study (East Nusa Tenggara and Sumatra), study participants may have had a higher-than-average awareness and understanding of dementia. Another issue to consider when interpreting our findings is that the larger study from which the present data were taken examined care networks of older people in Indonesia in general, rather than being focused on dementia. While this underlined the rarity with which the topics of (suspected) dementia or *pikun* were raised by family carers and healthcare volunteers, it limited the number of cases and volume of data on dementia for us to draw on. By not actively recruiting people living with dementia, our study may have especially under-sampled people with suspected dementia who live behind closed doors or alone with no carers.

As recommendations arising from our study, we argue for the development of more collaborative approaches to providing care for older people with suspected dementia and for initiatives in which healthcare practitioners and family members make the person with dementia their care partner. In terms of the recommendations to make care more collaborative, recently a successful home-visit pilot programme for older house-bound people was run in Yogyakarta, Indonesia (Schröder et al., 2024; 2023) . This intervention involved community health volunteers being trained on older people’s health problems, communication with older people, including those with suspected dementia, care practices, health and nutrition advice, conducting health checks and supporting family carers. While the intervention was not exclusively directed at older people with dementia, some of the recipients of home visits had suspected dementia. Older people appreciated the positive attention that the home visits provided, and family carers valued the advice, moral support, and recognition they received from volunteers. Especially in cities, where population densities are high, such schemes could be tailored specifically toward conducting home visits to older people with suspected dementia.

In terms of the second recommendation, a recent review of educational interventions for dementia carers in LMICs showed that interventions are promising (Evans et al., 2024), especially a multicomponent group intervention trialled in Egypt (Tawfik et al., 2021). Evans et al. (2024, p.1) argue that “Collaboration between LMICs, high-income countries (HICs), and carers is crucial in developing interventions tailored to meet carer needs whilst accounting for feasibility and equity for dementia care worldwide”. Family carers and those they care for can be encouraged to seek out dementia education, and key figures in their communities can support and refer them in this. Even where local or national educational initiatives are not currently available, there are free online resources, for example those developed by The Wicking Dementia Research and Education Centre, Australia, for “people in the early stages of the disease, their families and carers” (Eccleston et al., 2019; Farrow et al., 2022). Peer support groups also fit with Indonesian models of community activity and sociality and would strengthen familial and community ties around people with dementia. In addition, universities across multiple departments (Medicine, Nursing, Psychology, Public Health, Social Sciences) could add dementia to the curriculum to raise awareness and understanding. These kinds of educational, peer- and volunteer-based approaches strengthen rather than displace familial and community ties and fit with Indonesian cultural strengths and values.

## Conclusion

Awareness of and resources for dementia are limited in Indonesia. Daily care for people living with suspected dementia is carried out by family carers, with some support from relatives, neighbours and volunteers. Many people with dementia function relatively normal and stay engaged with activities and other people. They are often not recognized as having a health- or cognitive issue, but may receive physical, ADL, or monetary support in their homes. When care needs become sustained and complex, this can negatively impact on family carers. Once dementia affects people’s behaviour or personality, they may be stigmatized or kept behind locked doors. Professional dementia care in Indonesia is almost non-existent, but in specific locales knowledgeable volunteers exist. The presence of dementia-specific care seems to depend on a small number of people, be they family member, medical professional or volunteer, who have educated themselves about dementia and developed a more tailored way of communicating with and caring for people living with dementia. Recommendations include widening the pool of individuals with dementia understanding, promoting awareness and acceptance of those with more pronounced symptoms of dementia, and a more inclusive style of caregiving. At the same time, the strengths of Indonesian society, such as strong familial and societal ties, should be built upon to improve the lives of people living with dementia.

## Data Availability

Due to the sensitive nature of our ethnographic data and the risks of identifying communities and participants by making our anonymized data available, we have not deposited our data in any data repository.<

## Footnotes

1 The project was a collaboration between the University of Southampton, Universitas Katolik Indonesia Atma Jaya, Jakarta, Loughborough University and the University of Oxford.
